# U.S. state correlations between oral health metrics and Alzheimer’s disease mortality, prevalence and subjective cognitive decline prevalence

**DOI:** 10.1038/s41598-020-77937-8

**Published:** 2020-12-01

**Authors:** Rana X. Scherer, Warren J. Scherer

**Affiliations:** 1 University of Central Florida, The Burnett Honors College, 12778 Aquarius Agora Drive, Orlando, FL 32816-1800 USA; 2 St. Luke’s Cataract & Laser Institute, 43309 U.S. Highway 19 N., Tarpon Springs, FL 34689 USA

**Keywords:** Neuroscience, Neurology

## Abstract

Given the association between periodontal disease (PdD) and Alzheimer’s disease (AD), we examined correlations between states’ age-adjusted AD mortality rates, AD prevalence, subjective cognitive decline (SCD) prevalence, and oral health data. Data sources include the Centers for Disease Control and Prevention, scientific literature, and oral health rankings formulated by WalletHub.com and Toothbrush.org. Pearson (r) or Spearman (r_s_) correlation coefficients were generated and evaluated. AD mortality rates correlate with dental visits (r = − 0.50, p = 0.0003), partial (r = 0.39, p = 0.005) or total (r = 0.44, p = 0.001) edentulism, WalletHub.com (r_s_ = 0.30, p = 0.03) and Toothbrush.org (r_s_ = 0.35, p = 0.01) rankings. AD prevalence correlates with dental visits (r = − 0.30, p = 0.03), partial (r = 0.55, p = 0.00003) or total (r = 0.46, p = 0.0009) edentulism, prevalence of any (r = 0.38, p = 0.006) or severe-stage (r = 0.46, p = 0.0009) PdD, and WalletHub.com (r_s_ = 0.38, p = 0.006) rankings. SCD prevalence in adults aged ≥ 45 years correlates with dental visits (r = − 0.69, p < 0.00001), partial (r = 0.33, p = 0.02) or total (r = 0.37, p = 0.008) edentulism, prevalence of any (r = 0.53, p = 0.0001) or severe-stage (r = 0.57, p = 0.00002) PdD, WalletHub.com (r_s_ = 0.53, p = 0.00008) and Toothbrush.org (r_s_ = 0.60, p < 0.00001) rankings. State metrics indicative of compromised oral health correlate with AD mortality rates, AD prevalence and SCD prevalence.

## Introduction

Recently, evidence has emerged associating periodontal disease (PdD) with the development of, and cognitive decline in, Alzheimer’s disease (AD)^1–7^. Direct causation has not been definitively established; however, the preponderance of research points to a causative relationship between PdD and AD^8^. This comorbidity is consistent with the “antimicrobial protection hypothesis” of AD pathogenesis^9–11^. The hypothesis asserts that hallmark AD features, including extracellular amyloid-beta (Aβ) plaques, hyper-phosphorylation of tau protein, formation of intracellular neurofibrillary tangles, resultant neuroinflammation and neurodegeneration, are due to host responses to central nervous system (CNS) invasion by pathogens and/or their virulence factors^11–15^.

Key to the immune response to CNS infection is Aβ itself. Once thought to be an incidental catabolic byproduct without a normal physiological role, Aβ has been shown to function as an antimicrobial peptide, a component of the innate immune system^16^. In the CNS, Aβ binds and disrupts bacterial, viral, and fungal cell membranes, resulting in aggregation and agglutination of pathogenic organisms^17–22^. Investigations have shown that Aβ production is induced by exposure to pathogens, their virulence factors and/or chronic neuroinflammation^23–25^. Accordingly, Aβ represents an immediate and continued physiological response to invading microorganisms, stimulating chronic neuroinflammation and subsequent compromise of blood–brain-barrier function^25–27^. Over time, this innate immune response, fueled by continued exposure to bacterial virulence factors, chronic CNS infection, or CNS pathogen reactivation, serves to irreversibly prime microglia to switch into an aggressive, pro-inflammatory state^28–31^.

Consistent with age-dependency of AD development, CNS microglial responses to inflammatory challenge are similarly age-dependent. In young brains, microglial activation is associated with a reparative inflammatory response whereas in aged brains, an exacerbated inflammatory response occurs, resulting in proinflammatory microglial priming, neuronal death, synaptic loss, and cognitive decline^32,33^. Age-related, microglial-associated neurophysiological deficits have also been demonstrated by the inability to generate long-term potentiation, a cellular correlate for learning and memory, in the hippocampus of systemically-inflamed middle-aged, but not young rats^34^. Further supporting the role of microglia in this process, these investigators report that minocycline, an inhibitor of microglial activation, restored long term potentiation in systemically-inflamed middle-aged rats.

PdD is a nonreversible, chronic, infectious, systemic inflammatory condition characterized by gram-negative polymicrobial dysbiosis of the sub-gingival microbiome^28^. Within the subgingival pocket, colonization by causative organisms such as *Porphyromonas gingivalis*, *Tannerella forsythia* and *Treponema denticola* facilitates biofilm formation and PdD development^35,36^. The host’s exuberant systemic immunologic response to these pathogens results in destruction of gingival connective tissue, resorption of tooth-supporting alveolar bone and tooth loss^37,38^. Once PdD is established, dental procedures, brushing, flossing, or chewing can induce transient bacteremia, and planktonic dissemination of organisms and their shed outer membrane vesicles to distant organs and tissues, including the heart, liver, brain, coronary and peripheral arteries^39–44^. Specific to AD, *P.gingivalis* and its virulence factors, e.g. lipopolysaccharide and gingipains, which are cysteine-proteases associated with bacterial outer membrane and shed membrane vesicles, have been identified in AD brains and in cerebral spinal fluid of suspected AD patients^25,45^. Furthermore, serum antibody levels against *P. gingivalis* are increased in AD patients compared to non-AD controls^46,47^. Repeat episodes of PdD-induced bacteremia provide microorganisms and virulence factors with a hematogenous route to the CNS across a blood–brain barrier compromised by chronic inflammation, or alternatively, direct CNS invasion via the trigeminal nerve or olfactory tract^48–50^.

SCD is defined as a self-experienced, persistent decline in cognitive capacity relative to a previously normal status^51^. First described in 1982, SCD is one of the earliest detectable risk factors for AD development^52–55^. Although SCD patients exhibit normal responses to cognitive tasks, functional magnetic resonance imaging studies have revealed aberrant brain activation patterns and compensatory cortical hyperactivation compared to controls^56^. Similar to AD, SCD is associated with CNS Aβ deposition and amyloid status in SCD is predictive of self-perceived cognitive state, current cognition and future cognitive decline^57–60^.

Studies have confirmed comorbid relationships between AD prevalence, progression and severity, and PdD in select cohorts^2,4,7,46,61–65^. In a large cohort, Chen et al. recently discovered that patients with a 10 year history of PdD had a two-fold greater risk of developing AD compared to patients without PdD^7^. Unlike AD, little is known regarding the relationship between PdD and SCD; however, one small study has reported a positive correlation^66^. No previous study has analyzed associations between AD, SCD and PdD on a national level in the U.S. via comparisons of state-specific population data. The present study, utilizing state epidemiological data derived from governmental agencies, and scientific literature, is the first to examine U.S. state relationships between AD, SCD and oral health.

## Materials and methods

All methods were carried out in accordance with relevant guidelines and regulations. All experimental protocols were reviewed and approved by the Office of the Medical Directorate, St. Luke’s Cataract & Laser Institute, Tarpon Springs, FL. Because individual patients did not participate as subjects in this research, informed consent was not applicable to the present study. All third party data utilized in this study is available free of charge in the public domain from online databases and government websites (see references for each dataset).

For all 50 U.S. states, age-adjusted AD mortality, age-adjusted AD prevalence, SCD and oral health status data were obtained from several sources. Age-adjusted AD 2016 mortality rates were acquired from the Centers for Disease Control and Prevention (CDC) National Center for Health Statistics website^67^. Estimates of states’ age-adjusted AD prevalence were obtained from a study by Koller and Bynam which reviewed records of 4.8 million U.S. Medicare beneficiaries across all U.S. states^68^. State-specific SCD prevalence rates in adults aged ≥ 45 years were obtained from a study of 227,393 respondents across the U.S^69^. Because CDC oral health data and CDC age-adjusted AD mortality rates were subdivided by gender, a gender-based analysis for these data was also performed. All data used for analysis provided in spreadsheet format in Online Resource 1.

Oral health data reporting percentage of adults visiting a dental clinic and adults > 65 years who have lost all, or ≥ 6 teeth were acquired from the CDC Oral Health Data by Location which completes 400,000 adult interviews each year^70^. State PdD prevalence rates were obtained from a study by Eke et al. which utilized CDC data from the National Health and Nutrition Examination Survey and Behavioral Risk Factor Surveillance System^71^. Statistics regarding state water fluoridation rates were obtained from the Office of Disease Prevention and Health Promotion^72^.

Data from two national, non-peer-reviewed online state oral health ranking scales developed by WalletHub.com and Toothbrush.org were utilized for comparison to AD and SCD data^73,74^. The WalletHub.com scale, devised by 7 dental health researchers, evaluated state dental health across two key dimensions, “dental habits and care”, and “oral health”. These dimensions utilized 26 weighted metrics to generate composite U.S. state oral health scores. The Toothbrush.org ranking system evaluated state oral health status utilizing 20 metrics. Similar to WalletHub.com, the Toothbrush.org state rating system generated composite state oral health rankings based on “dental habits & care” and “oral health. In order to determine whether the WalletHub.com and Toothbrush.org rankings were consistent, a Bland Altman analysis was performed on the ranking data. Upper and lower limits of agreement were calculated by multiplying the standard deviation by (+) or (−) 1.96, respectively.

State-specific age-adjusted AD mortality rates, age-adjusted AD prevalence data and SCD prevalence data were compared to corresponding oral health data to determine whether there existed significant relationships between state AD, SCD and oral health data. Pearson (r) or Spearman rank (r_s_), correlation coefficients, were calculated and tested (two-tailed) for statistical significance (p-value ≤ 0.05) against the null hypothesis of no correlation, *H*_0_: ρ = 0 using a standard table of critical values for r. Ranked data from WalletHub.com and Toothbrush.org were evaluated using Spearman rank correlation coefficients. Microsoft Excel was used to calculate correlation coefficients and generate scatter plots.

It has been reported by some authors that the Pearson correlation is robust and can withstand violations of assumptions such as normality whereas others state that highly non-normal data distributions can reduce r values and inflate Type I error rates^75^. Given that non-normal data distributions could potentially affect r, a Kolmogorov–Smirnov test statistic (D) was calculated for each data set to determine whether it is normally distributed. A corresponding p-value ≤ 0.05 indicates that the data set is not normally distributed.

## Results

The Kolmogorov–Smirnov test was performed on all Pearson-analyzed data sets to access normality. The age-adjusted AD mortality rates (D = 0.086*; p* value = 0.73), age-adjusted AD prevalence (D = 0.094*; p* value = 0.83), and SCD prevalence (D = 0.107*; p* value = 0.58) data sets were all found to be normally distributed. The CDC oral health survey data sets addressing the percentage of adults who had visited a dentist during the previous year (D = 0.081*; p* value = 0.88), the percentage of adults > age 65 years who had lost all of their teeth (D = 0.121*; p* value = 0.42), and the percentage of adults > age 65 years who had lost ≥ 6 teeth (D = 0.099*; Pp* value = 0.67) were found to be normally distributed. The U.S. all-stage PdD prevalence (D = 0.095*; p* value = 0.73) and severe-stage PdD prevalence (D = 0.094*; p* value = 0.73) data sets were found to be normally distributed. In summary, all data sets utilized for Pearson correlation coefficient analysis in the present study were found to be normally distributed.

**Figure 1:**
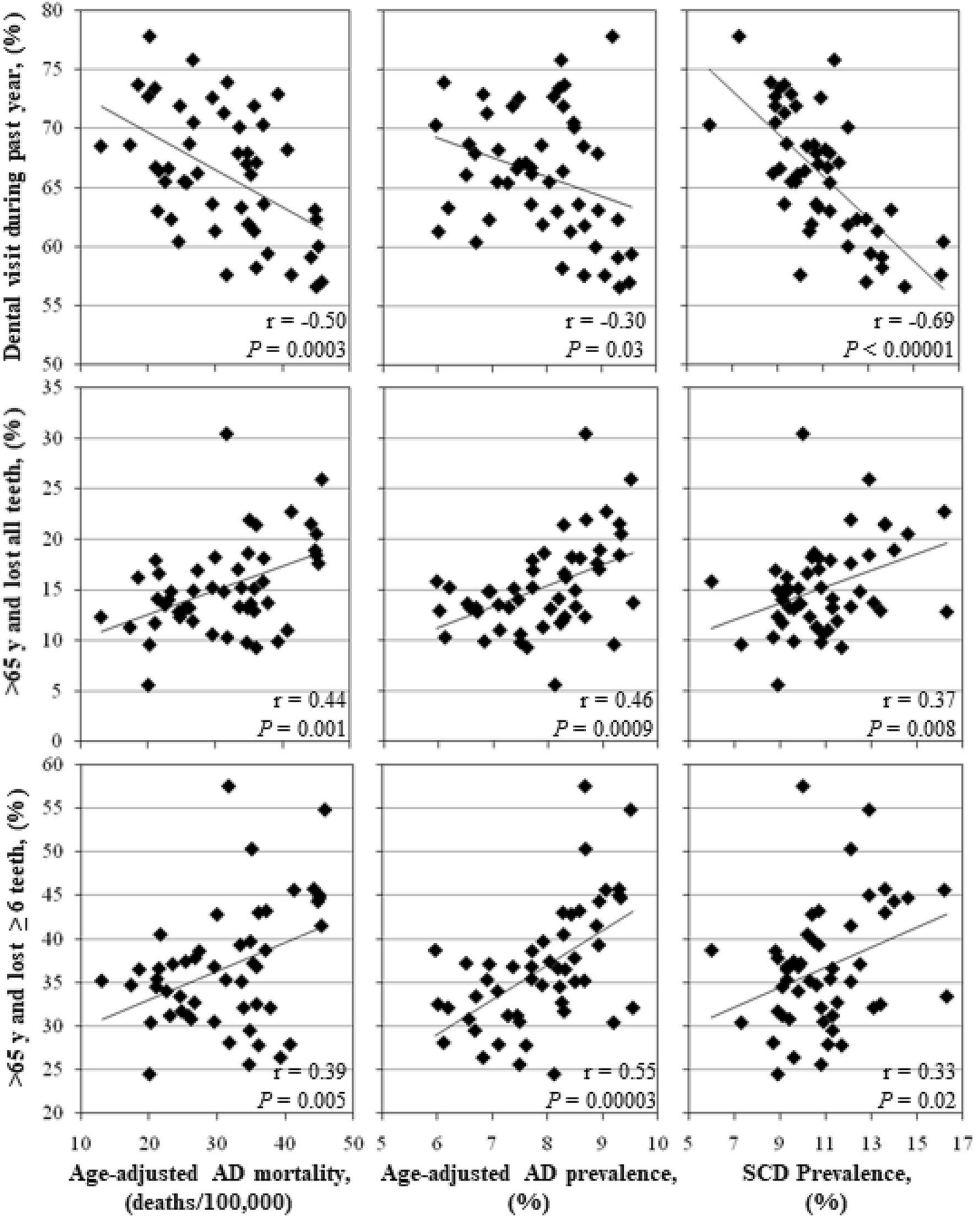
CDC dementia data v CDC oral health metrics. Scatter-plots showing correlations between state-specific age-adjusted AD mortality rates, age-adjusted AD prevalence, SCD prevalence and CDC data regarding state population percent visiting a dentist during the past year, and percentage of adults > 65 years who have lost all or ≥ 6 teeth. Each point represents data from one U.S. state. The Pearson correlation coefficient (r) and corresponding *P*-value are presented for each correlation. *AD* Alzheimer’s disease, *SCD* subjective cognitive decline, *CDC* Centers for Disease Control and Prevention.

For each state, 2016 CDC oral health survey data addressed the percentage of adults who had visited a dentist during the previous year, the percentage of adults > age 65 years who had lost all of their teeth, and the percentage of adults > age 65 years who had lost ≥ 6 teeth (Table 1, Fig. 1). There were significant negative correlations between the percentage of adults who visited a dentist and age-adjusted AD mortality rates (r = − 0.50*; p* value = 0.0003), age-adjusted AD prevalence (r = − 0.30*; p* value = 0.03), and SCD prevalence (r = − 0.69*; p* value < 0.00001). There were significant positive correlations between the percentage of adults > age 65 years who had lost all of their teeth and age-adjusted AD mortality rate (r = 0.44*; p* value = 0.001), age-adjusted AD prevalence (r = 0.46*; p* value = 0.0009), and SCD prevalence (r = 0.37*; p* value = 0.008). Similarly, there were also significant positive correlations between the percentage of adults > age 65 years who had lost ≥ 6 teeth and age-adjusted AD mortality rate (r = 0.39*; p* value = 0.005), age-adjusted AD prevalence (r = 0.55*; p *value = 0.00003), and SCD prevalence (r = 0.33*; p* value = 0.018).

**Table 1:** U.S. state-specific age-adjusted AD mortality rates, age-adjusted AD prevalence, SCD prevalence vs. oral health parameters.

Oral Health Data	Age-adjusted AD mortality rate		Age-Adjusted AD Prevalence		SCD Prevalence (≥ 45 years)	
	r	*p*-value	r	*p*-value	r	*p*-value
**% visiting dentist, past year**						
all adults	− 0.50	0.0003	− 0.30	0.03	− 0.69	< 0.00001
females	− 0.52	0.0001				
males	− 0.42	0.003				
**% lost all teeth, > 65 years**						
all adults	0.44	0.001	0.46	0.0009	0.37	0.008
females	0.43	0.001				
males	0.37	0.007				
**% lost ≥ 6 teeth, > 65 years**						
all adults	0.39	0.005	0.55	0.00003	0.33	0.02
females	0.36	0.009				
males	0.34	0.02				
All-stage PdD^a^ prevalence	0.08	0.57	0.38	0.006	0.53	0.0001
Severe-stage PdD prevalence	0.15	0.30	0.46	0.0009	0.57	0.00002
WalletHub.com oral health overall ranking^b^	0.30	0.03	0.38	0.006	0.53	0.00008
Toothbrush.org oral health overall ranking^b^	0.35	0.01	0.25	0.08	0.60	< 0.00001
% Drinking water fluoridation	0.09	0.55	0.07	0.63	− 0.01	0.99

*AD* Alzheimer’s disease, *SCD* Subjective cognitive decline.

^a^Periodontal disease.

^b^r = Spearman correlation coefficient for ranked data (r_s_).

Gender-based, age-adjusted AD mortality rate data showed similarly significant correlations with corresponding oral health data regarding percentage of females (r = − 0.52*; p* value = 0.0001) or males (r = − 0.42*; p* value = 0.002) visiting a dentist or dental clinic during the previous year, percentage of females (r = 0.43*; p* value = 0.001) or males (r = 0.37*; p* value = 0.007) > age 65 years who have lost all their teeth, and percentage of females (r = 0.36*; p* value = 0.009) or males (r = 0.34*; p* value = 0.02) > age 65 years who had lost ≥ 6 teeth (Table 1).

**Figure 2:**
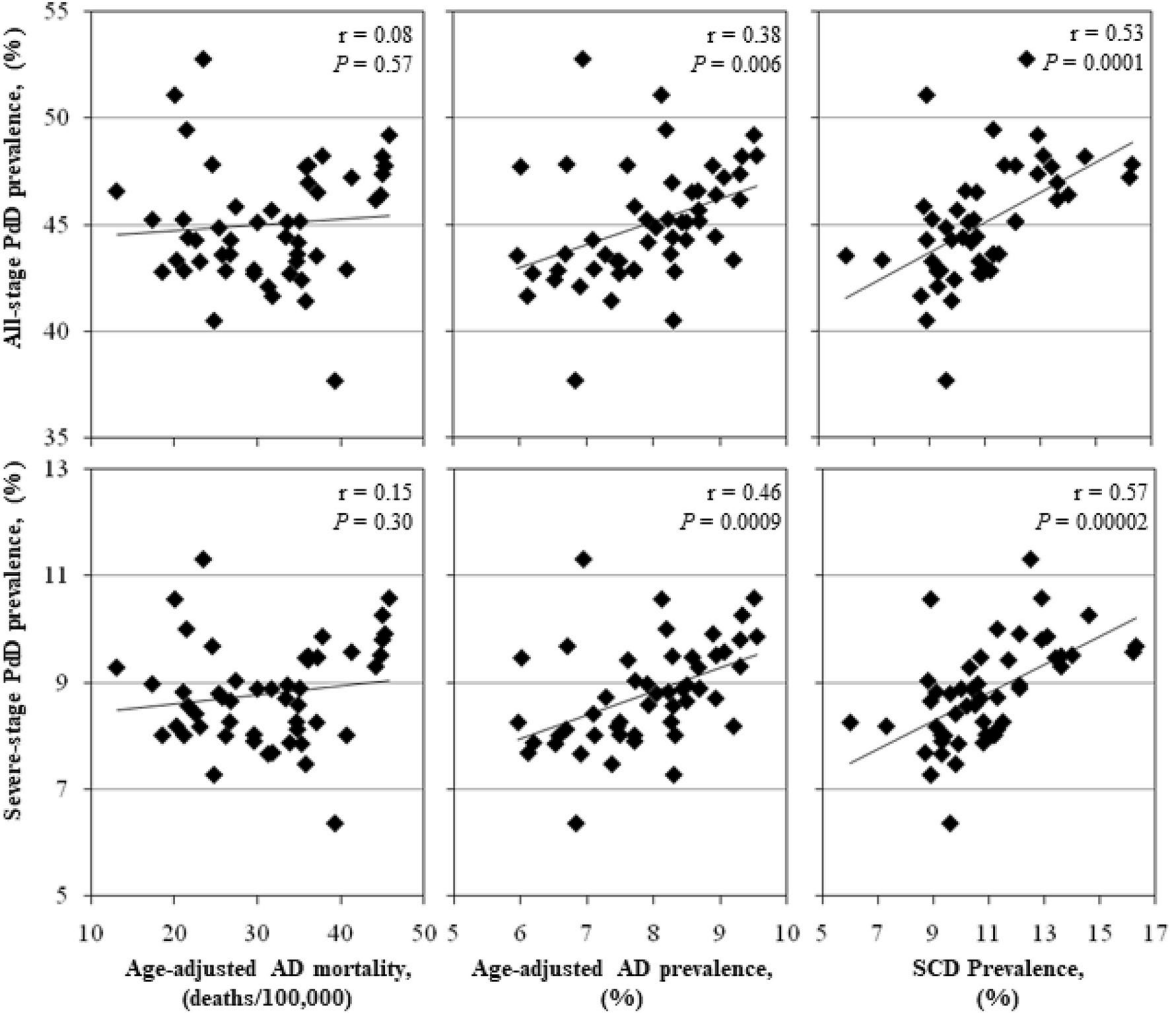
CDC dementia data v PdD prevalence data. Scatter-plots showing correlations between state-specific age-adjusted AD mortality rates, age-adjusted AD prevalence, SCD prevalence and state PdD prevalence data for all-stages of PdD and severe-stage PdD. Each point represents data from one U.S. state. The Pearson correlation coefficient (r) and corresponding *P*-value are presented for each correlation. *AD* Alzheimer’s disease, *SCD* subjective cognitive decline, *PdD* periodontal disease, *CDC* Centers for Disease Control and Prevention.

Associations between all-stage PdD prevalence and severe-stage PdD prevalence were compared to age-adjusted AD mortality rate, age-adjusted AD prevalence and SCD prevalence (Table 1 and Fig. 2). No significant correlation was found between age-adjusted AD mortality rate and all-stage PdD prevalence (r = 0.08*; p* value = 0.56) or severe PdD prevalence (r = 0.15*; p* value = 0.30). Significant correlations were found between total PdD prevalence and age-adjusted AD prevalence (r = 0.38*; p* value = 0.006) and SCD prevalence (r = 0.53*; p* value = 0.0001). Significant correlations were found between severe PdD prevalence and age-adjusted AD prevalence (r = 0.46*; p* value = 0.0009) and SCD prevalence (r = 0.57*; p* value = 0.00002).

**Figure 3:**
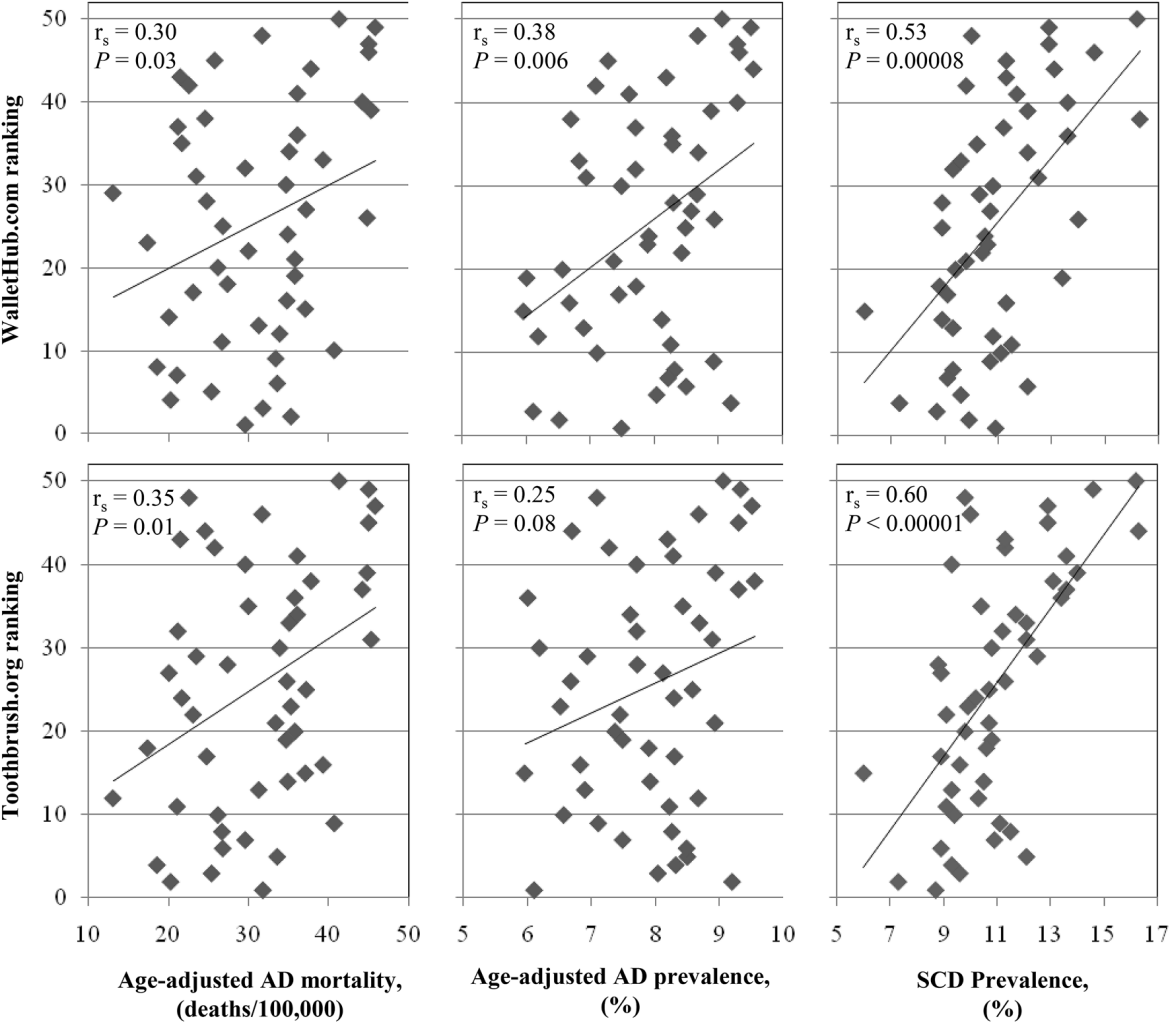
CDC dementia data v State oral health ranks. Scatter-plots showing correlations between state-specific age-adjusted AD mortality rates, age-adjusted AD prevalence, SCD prevalence and WalletHub.com and Toothbrush,org state oral health rankings. Each point represents data from one U.S. state. The Spearman correlation coefficient (r_s_) and corresponding *p*-value are presented for each correlation. *AD* Alzheimer’s disease, *SCD* subjective cognitive decline, *CDC* Centers for Disease Control and Prevention.

**Figure 4:**
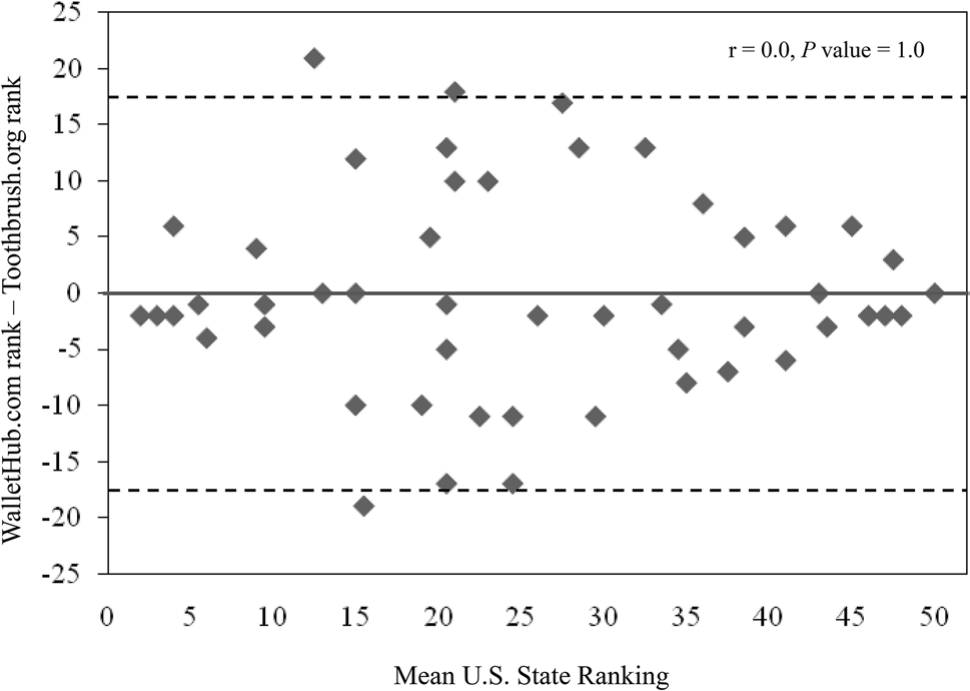
Bland Altman analysis comparing Toothbrush.org and WalletHub.com oral health ranking systems. No significant differences (r = 0, *p*-value = 1.0) were found between the WalletHub.com and Toothbrush.org ranking systems. Dotted lines represent 95% upper (17.56) and lower (− 17.56) limits of agreement. Bias = 0, S.D. =  ± 8.96.

WalletHub.com and Toothbrush.org U.S. state oral health rankings were compared to respective age-adjusted AD mortality rate, age-adjusted AD prevalence and SCD prevalence (Table 1, Fig. 3). The WalletHub.com total oral health rankings were found to be significantly correlated with age-adjusted AD mortality rate (r_s_ = 0.30*; p* value = 0.03), age-adjusted AD prevalence (r_s_ = 0.38*; p* value = 0.006) and SCD prevalence (r_s_ = 0.53*; p* value = 0.00008). The Toothbrush.org total oral health rankings were significantly correlated with age-adjusted AD mortality rate (r_s_ = 0.35*; p* value = 0.01) and SCD prevalence (r_s_ = 0.60*; p* value < 0.00001), however, the correlation with age-adjusted AD prevalence (r_s_ = 0.25) did not reach significance (*p* value = 0.08). In order to determine whether these two state-by-state ranking systems were consistent with one another, a Bland Altman analysis was performed and revealed no significant differences (r = 0, *p*-value) between the Toothbrush.org and WalletHub.com state rankings (Fig. 4).

Drinking-water fluoridation, which reduces dental caries but is ineffective against PdD, was utilized as a negative control and examined as a factor associated with AD and SCD (Table 1). No significant correlations were found between the percentage of each states’ population served by fluoridated drinking water and age-adjusted AD mortality rate (r = 0.09*; p* value = 0.60), age-adjusted AD prevalence (r = 0.07*; p* value = 0.63) or SCD prevalence (r = − 0.003*; p* value = 0.99) (Table 1). Additionally, no correlations were found between the percentage of each states’ population that is served by fluoridated drinking water and all-stage PdD prevalence (r = − 0.04*; p* value = 0.81) or severe stage PdD prevalence (r = 0.005*; p* value = 0.97), confirming that state water fluoridation rates are unrelated to PdD prevalence.

## Discussion

Utilizing state-specific, epidemiologic data, we conducted a nationwide correlational analysis examining possible associations between age-adjusted AD mortality rates, age-adjusted AD prevalence, SCD prevalence and oral health metrics relevant to PdD in the U.S. The rationale underlying these comparisons stems from the “antimicrobial protection hypothesis” of AD and recent investigations linking CNS infection, particularly the chronic, recurring infection that is pathognomonic of PdD, with the development and progression of AD.

All correlations between CDC oral health data and dementia metrics were statistically significant; however, the strongest association existed between SCD prevalence and the percentage of respondents who visited a dental clinic during the previous year. This finding indicates that regular dental care is associated with a lower SCD prevalence among adults ≥ 45 years; however, it is not clear if the underlying cause of the association is due specifically to a reduction in the severity of PdD. Future research comparing oral health habits and dental health visits among SCD patients diagnosed with PdD versus controls could provide additional information. Given that SCD patients are generally younger than AD patients, establishment of a causative link between SCD and PdD could dramatically impact future medical and economic burdens of AD via policies and programs that address timely, routine dental prophylaxis.

It is known that PdD is the main cause of tooth loss in middle aged and older adults^76^. With regard to tooth loss and edentulous condition among patients older than 65 years, the strongest correlations were observed with age-adjusted AD mortality rates and age-adjusted AD prevalence compared to SCD prevalence. This finding is not unexpected as the AD data comparison reflects a similar age group (i.e.; adults aged 65 + years) compared to the SCD prevalence data (adults ≥ 45 years). These findings are in general agreement with previous studies regarding AD and tooth loss^77^. Stein et al. found that tooth loss due to PdD doubles the risk of onset of AD^78^. A recent meta-analysis performed by Dioguardi et al. reported results with increased rates of partial tooth loss (hazard ratio: 1.52) and complete edentulous condition (hazard ratio: 2.26) amongst AD patients compared to controls^79^. These finding have been supported by other cohort studies. Specifically, the Hisayama study reported correlation between the loss of dental elements and dementia caused specifically by AD, as significant correlations were not found for dementia of vascular origin^80^. Additionally, the simple absence of teeth did not impact Aβ levels or hippocampal pyramidal cells in transgenic AD model mice whose molar teeth were surgically extracted^81^. With regard to gender and tooth loss, we found similar, significant correlations between age-adjusted AD mortality rates and CDC oral health data for both males and females.

State prevalence of any-stage PdD, and severe-stage PdD were found to correlate significantly with both age-adjusted AD prevalence and SCD prevalence. However, no significant associations were found between any-stage or severe-stage PdD prevalence and age-adjusted AD mortality rates. A plausible explanation for this seemingly contradictory finding may be due to confounding effects of *P. gingivalis* and other PdD pathogen-induced mortality from diseases of organ systems other than the CNS. For example, it has been established that in addition to brain, *P. gingivalis* can migrate to other systemic sites, such as the cardiovascular system and liver^40,41^. Several studies have reported the presence of PdD pathogens in coronary atheromatous plaques from myocardial infarction patients and all clinically normal areas of coronary and femoral arterial wall samples from patients with a history cardiovascular disease were found to be colonized by *P gingivalis*^42,82^. Furthermore, viable PdD pathogens have been cultured from atherosclerotic plaques^83^. Clinical and epidemiological studies have demonstrated that PdD contributes to increased mortality from heart failure, ischemic heart disease, peripheral vascular disease, stroke, cancer and pneumonia^84–92^. All of these conditions are also common in elderly patients afflicted with AD. Therefore, mortality due to myocardial infarction, stroke, cancer or pneumonia in a mildly or moderately affected AD patient would necessitate listing the official “cause of death” as an entity other than AD. This reasoning may help to explain why AD prevalence, but not AD mortality, is correlated with PdD prevalence.

The strongest state-by-state correlations existed between PdD (all-stage or severe-stage) prevalence and SCD prevalence. Because active PdD has been shown to result in continued cognitive decline in AD, aggressive PdD treatment may help to slow or reverse cognitive changes in younger patients with SCD. Once approved, another potential option could include treatment with oral gingipain inhibitors which have been shown to reduce CNS bacterial load, block Aβ production, reduce neuroinflammation, and rescue hippocampal neurons in a tissue culture and mouse model^25^.

Although not specific for PdD, U.S. state rankings from two independent oral health evaluation systems formulated by WalletHub.com and Toothbrush.org were compared to corresponding age-adjusted AD mortality rates, age-adjusted AD prevalence and SCD prevalence. There were significant correlations between WalletHub.com composite state oral health rankings and age-adjusted AD mortality rates, age-adjusted AD prevalence and SCD prevalence. Similarly, significant correlations were found between Toothbrush.org composite state oral health rankings and respective age-adjusted AD mortality rates and SCD prevalence. Given the multi-factorial parameters incorporated into these oral health rating systems, the significance of the associations between state oral health scores and corresponding AD and SCD state epidemiological data is remarkable. Similar state-based ranking scales including factors more specific to PdD-related oral health such as tooth loss, bleeding on probing testing, periodontal pocket depth and degree of gingival recession might be expected to generate even stronger correlations with AD and SCD epidemiological data.

The association between water fluoridation and age-adjusted AD mortality rates, AD prevalence and SCD prevalence was also examined. The addition of fluoride to public water supplies acts to reduce tooth decay and does not impact PdD^93^. The percent of state populations receiving fluoridated water varies across the U.S. We found no correlation between the percent of each state’s population served by water fluoridation and corresponding state AD or SCD data. This finding supports the hypothesis that the correlations between oral health metrics and AD data observed in this study are specific to PdD rather than other dental conditions such as caries.

There are limitations of the present study. Due to the disparate nature and sources of the data sets, it was impossible to control for, or adjust for all possible confounding variables, such as economic or educational status, medical comorbidities, ethnicity or age. In certain cases, similarly aged groups were directly compared (e.g.: CDC data regarding edentualism in adults aged 65 + v. AD mortality or prevalence) whereas in other cases, the dataset referred to adults of all-ages (e.g.: CDC data reporting percentage of adults visiting a dentist). Unfortunately, datasets adjusted for all possible confounding variables are simply not available. However, given the levels of significance of our findings, we contend that the adult datasets from high-risk (high mortality and/or prevalence) AD states represent populations that are at greater risk for developing AD or cognitive decline later in life.

Other limitations include the inability to conclusively attribute a clinical causation between PdD and AD, limited sample sizes of cross-sectional datasets relative to state populations, and possible inherent biases of the Toothbrush.org and WalletHub.com oral health rankings. Agreement between the results of the Toothbrush.org and Wallethub.com ranking scales based on Bland Altman analysis obviates some concerns regarding bias of either ranking system. Additionally, CDC data are self-reported and could be biased or inaccurate due to regional differences in survey-response behavioral patterns. However, the sampling of separate cohorts (i.e.: all adults v adults > 65 years and variability of questions (e.g.: dental visits v tooth loss), produced responses that consistently associate AD and SCD with poor oral health. Given these circumstances, it is unlikely that the correlations observed between AD data and CDC oral health data are due solely to regionally-related survey response bias. It is established that PdD is the main etiology underlying tooth loss in middle-aged and elderly adults, however, CDC oral health-related data sets would necessarily include other oral health-related etiologies of tooth loss in addition to PdD^72^. These conditions include trauma, caries, xerostomia, and chronic enamel erosion. One would expect that inclusion of non-PdD-related conditions would serve to confound, dilute and decrease associations between PdD and AD or SCD. However, despite the inability to exclude PdD-unrelated oral conditions from data sets, strong associations with AD and SCD data were still demonstrated. Additionally, several PdD-associated conditions such as stroke, ischemic heart disease, heart failure and peripheral vascular disease are also frequently encountered in AD patients. The presence or absence of any of these conditions could conceivably confound the results of the present study. Unfortunately, it was not possible to consider these effects as the cross-sectional epidemiological data available for this study did not include information on these comorbid conditions.

In conclusion, significant correlations between state age-adjusted AD mortality, age-adjusted AD prevalence, SCD prevalence and statewide indicators of poor oral health in the U.S were found. Because PdD is a modifiable risk factor, government and private insurance programs or initiatives that positively impact PdD prevalence and severity could substantially reduce the clinical, social and financial burden of AD.

## Supplementary information

Supplementary Information.

## Data Availability

All data used available in public domain online. Original spreadsheet for data analysis submitted as supplementary material.
